# Evolution and trends in the management of acromioclavicular joint dislocations: an epidemiological analysis from Germany between 2013 and 2023

**DOI:** 10.1016/j.jseint.2025.03.005

**Published:** 2025-04-01

**Authors:** Philipp Herrmann, Martin Luedemann, Thilo Lehmeyer, Sebastian Frischholz, Maximilian Rudert, Kilian List, Ioannis Stratos, Felix Hochberger

**Affiliations:** Department of Orthopaedic Surgery, Julius-Maximilians University Wuerzburg, Koenig-Ludwig-Haus, Wuerzburg, Germany

**Keywords:** Shoulder surgery, Arthroscopic stabilization, Hospitalization, Demographic analysis, Acromioclavicular injuries, Epidemiology, AC joint dislocation

## Abstract

**Objective:**

This study aims to analyze nationwide trends in the management of hospitalized patients with acromioclavicular joint dislocations (ACJDs) in Germany over the past decade, focusing on surgical procedures and demographic patient data. The investigation examines the shift from conventional open stabilization techniques to mini-open and arthroscopically assisted approaches.

**Materials and methods:**

Hospital billing data from the Federal Statistical Office of Germany (2013-2023) were analyzed, including patients diagnosed with ACJD (International Classification of Diseases-10: primary diagnosis International Classification of Diseases S43.1) who underwent surgical reconstruction of the ACJD (German Operation and Procedure Classification System codes: 5-814b, 5-807.4, 5-807.5, 5-807.6, 5-930.0, 5-852.f, and 5-852.f8). Data included year of diagnosis, patient sex, surgical codes, and age (grouped in 5-year intervals). Data processing used R software for transformation and Tableau Desktop for subgroup analyses, stratified by type of procedure type, sex, and age. Statistical analyses, including linear regression and chi-squared tests, were conducted using GraphPad Prism (GraphPad Software, Inc., San Diego, CA, USA). Statistical significance was set at *P* ≤ .05.

**Results:**

From 2013 to 2023, 82,254 patients were hospitalized and surgically treated for ACJD in Germany, indicating a decline in inpatient surgical management (*P* < .05). This reduction primarily reflects a decrease in the number of inpatient procedures rather than changes in hospitalization duration or a shift toward outpatient surgery. Arthroscopically assisted stabilization increased from 19.6% in 2013 to 37.5% in 2023, becoming the most common surgical technique, while open techniques, such as plate stabilization and screw or Kirschner wire fixation, significantly decreased (*P* < .05). Tendon augmentation procedures showed a notable rise despite their rarity (*P* < .005). Arthroscopic techniques were predominantly used in younger patients (mean age: 39.6 years), while open reduction with plates was the most commonly used method in the age group over 60 years (*P* < .05). ACJD incidence was 8.2 times higher in men than women, with significant male dominance across all surgical techniques (*P* < .05).

**Conclusions:**

Over the past decade, the management of ACJD s in Germany has shifted toward arthroscopically assisted stabilization, particularly in younger patients, while traditional open techniques have declined. Numbers of hospitalized treated cases have decreased, with a strong male predominance and age-related preferences influencing surgical approaches.

Acromioclavicular joint dislocation (ACJD) is a common injury, particularly among young males, and accounts for 3%-12% of all shoulder injuries.^11^^,^^13^ The Rockwood classification system provides a framework for categorizing these injuries, ranging from type 1 to type 6, based on the degree of dislocation severity. Management strategies are largely guided by the Rockwood grade. Over time, numerous surgical approaches have been developed, with over 150 techniques described since the introduction of the Cadenet stabilization method for ACJD.^6^ While nonoperative treatment is widely accepted for Rockwood type 1 and 2 injuries, the optimal approach for type 3 and 5 injuries continues to be debated.^19^ Presently, most Rockwood type 3 injuries are initially managed conservatively, whereas surgical intervention is typically advised for type 5 dislocations.^5^^,^^6^^,^^19^^,^^35^^,^^42^ Several studies, including those with evidence levels 1 and 2, as well as numerous lower-level evidence studies, have suggested a preference for nonoperative management in patients with high-grade Rockwood injuries (types 3 and 5).^5^^,^^7^^,^^8^^,^^22^^,^^24^^,^^28^^,^^33^^,^^42^ A recent Cochrane review, analyzing 6 small trials, found that surgical intervention might not provide significant advantages in terms of functional outcomes, return to sports, or quality of life for patients with acute high-grade Rockwood injuries. However, these conclusions were based on low-quality evidence.^41^ Beyond the ongoing debate over nonoperative versus surgical management of specific Rockwood subtypes, the spectrum of surgical options for ACJD has broadened beyond conventional open stabilization procedures to include mini-open approaches and arthroscopically assisted techniques. Despite extensive research, no single surgical technique has been definitively established as superior in achieving optimal radiological, clinical, or functional outcomes, or in minimizing recurrence instability rates.^23^ Moreover, there is currently a lack of sufficient data to determine whether demographic patient factors may influence the preference for specific surgical techniques. To date, no comprehensive studies have investigated nationwide trends in the number of hospitalized treated ACJD cases, with specific consideration of the surgical techniques utilized over an extended period. Therefore, this study aims to provide a comprehensive analysis of current trends in the management of hospitalized patients with ACJD in Germany over the past decade, incorporating demographic patient data and the surgical procedures performed. Drawing on the existing body of evidence, the authors hypothesize a reduction in the overall number of surgical interventions and hospitalizations, accompanied by a gradual shift from traditional open stabilization techniques to arthroscopically assisted approaches.

## Materials and methods

### Data source and data structure

For this study, hospital billing data for patients were obtained from the Federal Statistical Office of Germany, covering the period from 2013 to 2023. The inclusion criteria were limited to patients diagnosed with ACJD, identified by the International Classification of Diseases (ICD-10) diagnosis code S43.1 as a primary diagnosis for the treatment in the hospital. Hospitalization in this study refers strictly to inpatient admission for surgical treatment, irrespective of the duration of hospital stay. Additionally, eligible patients must have undergone surgical procedures related to the acromioclavicular joint (ACJ) during the same hospital admission, as defined by the German Operation and Procedure Classification System (OPS) codes 5-814b (arthroscopic stabilization of the ACJ using fixation methods), 5-807.4 (open surgical suture of the ligaments of the clavicle), 5-807.5 (open surgical suture of the ligaments of the clavicle with plate stabilization), and 5-807.6 (open surgical suture of the ligaments of the clavicle with screw or wire fixation). The OPS code 5-807.4 refers to the open surgical suture of the ligaments of the clavicle, which primarily addresses direct ligament repair. In contrast, open reconstruction using suture cerclage (OSC) refers to a stabilization technique where sutures encircle structures, potentially including the coracoid process, to enhance stability. This study does not differentiate between these subtypes, as both procedures are classified under the same OPS code. However, it is generally assumed that both procedures provide equivalent biomechanical stability.^2^ For this study, ‘hospitalized patients’ refers to those who were admitted for inpatient care and underwent surgical intervention during their hospital stay. ‘Operated patients’ refer to all individuals who underwent ACJD-related surgery, regardless of inpatient or outpatient status. ‘Hospitalization’ in this context refers exclusively to inpatient cases recorded in hospital billing data. ‘Ambulatory surgeries’ denote procedures performed without hospital admission, which are not captured in our dataset. For cases of chronic instability of the acromioclavicular joint, the required surgical procedures were identified by OPS codes 5-930.0 (harvesting of autologous graft), 5-852.f (harvesting of tendon tissue for transplantation), or 5-852.f8 (harvesting of tendon tissue for transplantation from thigh or knee). This study focuses exclusively on hospitalized patients who underwent surgical intervention for ACJD. The dataset does not include outpatient cases or surgeries performed in ambulatory settings. While ACJD procedures may occasionally be performed on an outpatient basis, these cases were not captured in our data. The data were provided by an authorized representative of the Federal Statistical Office of Germany, specifically requested and procured for the purposes of this research. The dataset includes key parameters such as the year of diagnosis (2013-2023), patient sex (male or female), relevant OPS codes, and patient age that are categorized into 5-year intervals. URL: https://github.com/ioannis-stratos/acj.

### Data processing

In this study, data transformation from wide format to long format was conducted using R software (R-Studio version 2023.12.0, Posit, Boston, MA, USA) in conjunction with the 'reshape2′ package. Subgroup analyses were performed based on specific OPS codes: 5-814b for arthroscopically assisted ACJ stabilization (Arthro), 5-807.4 for OSC, 5-807.5 for open reduction and internal fixation with plate (ORIF-P) stabilization , and 5-807.6 for open reduction and internal fixation with screws or Kirschner wires (ORIF-S/K). For chronic instability of the acromioclavicular joint, subgrouping was based on procedures involving reconstruction using tendon augmentation (RTA), identified by OPS codes 5-930.0, 5-852.f, and 5-852.f8. Further stratification was performed by patient sex, year of diagnosis, and age, with age divided into 6 categories: “>20,” “21-30,” “31-40,” “41-50,” “51-60,” and “>60” years. Subgroup computations and analyses were carried out using Tableau Desktop (version 2023.3; Tableau Software, Seattle, WA, USA). The results were then organized into structured tables for detailed review and interpretation.

### Statistical analysis

Linear regression analyses were performed using GraphPad Prism software (version 10.1.1; GraphPad Software, San Diego, CA, USA), which was also utilized to create the corresponding graphical outputs. The F-test was applied to assess the overall significance of the regression models, determining whether they significantly deviated from the null hypothesis of no effect. In all analyses, the independent variable was time (2013-2023), represented on the x-axis, while the dependent variable was consistently plotted on the y-axis. For nonlinear regression calculations, Gaussian distributions were employed. The differences in group distributions were statistically analyzed using the chi-squared test. A *P* value threshold of ≤.05 was used to define statistical significance across all analyses.

## Results

### Overall results

**Figure 1:**
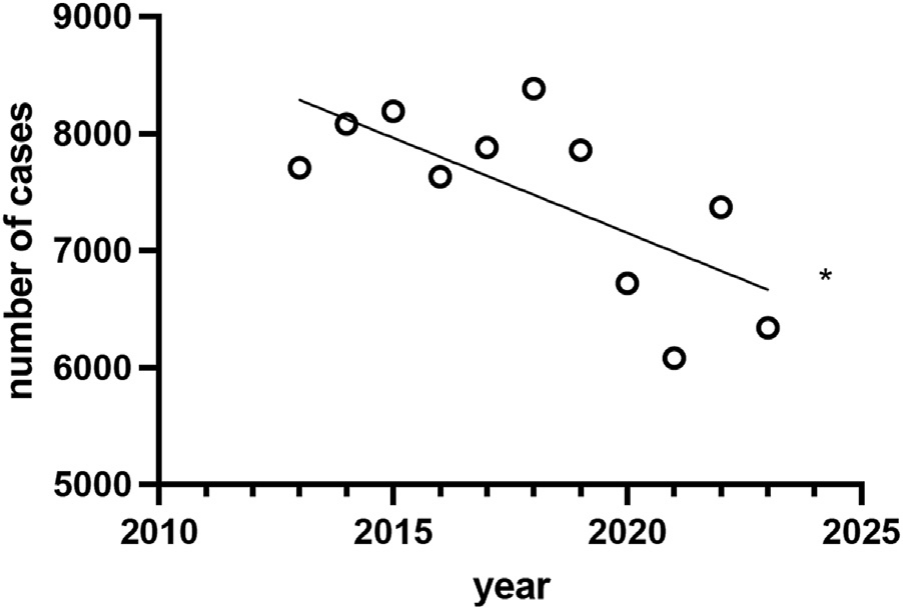
Linear regression analysis of the annual number of hospitalized patients diagnosed with ACJD (ICD-10 code S43.1) from 2013 to 2023. The regression demonstrates a statistically significant decline over the decade (R^2^ = 0.49, *P* < .05). *ICD*, International Classification of Diseases; *ACJD*, acromioclavicular joint dislocation.

**Figure 2:**
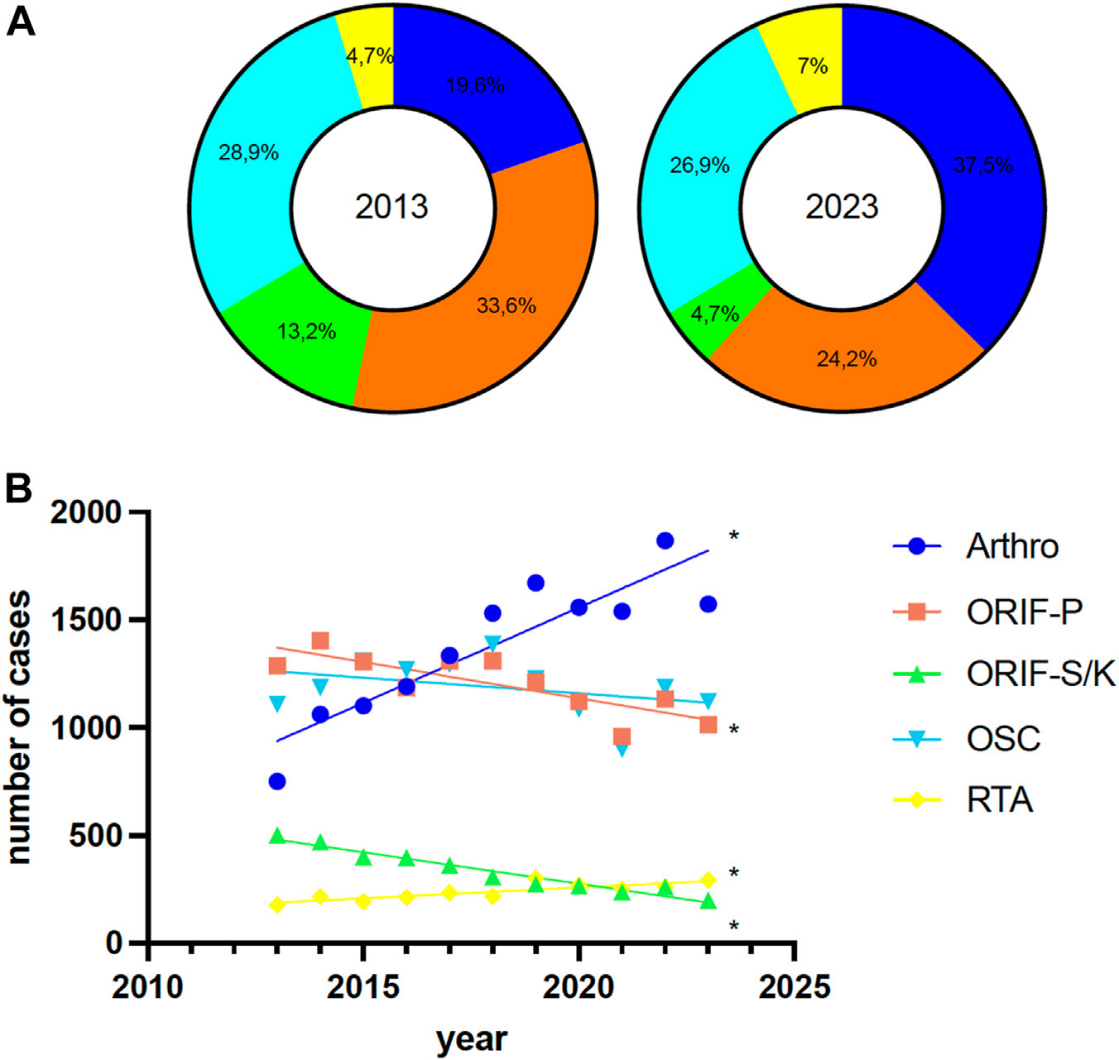
(**A**) Schematic representation of the relative distribution of surgical procedures for ACJD in 2013 and 2023, illustrating the shift from open to arthroscopic techniques. (**B**) Linear regression analysis of trends in specific surgical procedures over time, showing significant increases or decreases in their usage (eg, *Arthro*, arthroscopically assisted stabilization; *ORIF-P*, open reduction internal fixation with plate; *ORIF-S/K*, open reduction internal fixation with screw or Kirschner wire; *OSC*, open reconstruction using suture cerclage; *RTA*, reconstruction using tendon augmentation).

Between 2013 and 2023, a total of 82,254 patients were hospitalized in Germany due to ACJD. Over this period, a significant decline in the number of hospitalized patients was observed, which primarily reflects a decrease in inpatient surgical management rather than a shift toward nonoperative treatment (Fig. 1) (R^2^: 0.49; y = −162.1x + 334,669; F: 8.648; slope with *P* < .05 nonzero). Focusing on the various surgical procedures, a significant increase in the use of arthroscopically assisted ACJ stabilization was noted throughout the study period (2013: n = 751; 2023: n = 1572; R^2^: 0.82; y = 88.31x-176,828; F: 39.85; slope with *P* < .05 nonzero). The relative ratio of arthroscopically assisted ACJ stabilization rose from 19.6% in 2013 to 37.5% in 2023, making it the most performed surgical technique for treating ACJD in recent years (Fig. 2). In 2013, the most frequently used surgical technique was open stabilization with plate, with a relative ratio of 33.6%. However, by 2023, this proportion had decreased to 24.2%. Interestingly, both open surgical techniques with plate stabilization (2013: n = 1285; 2023: n = 1014; R^2^: 0.67; y = −33.60x + 69,008; F: 17.89; slope with *P* < .05 nonzero) and screw/Kirschner wire fixation (2013: n = 503; 2023: n = 199; R^2^: 0.95; y = −29.16x + 59,187; F: 163.5; slope with *P* < .05 nonzero) demonstrated a significant declining trend. In contrast, for OSC, no statistically significant difference was observed, though a downward trend was noted (2013: n = 1107; 2023: n = 1120; R^2^: 0.14; y = −14.50x + 30,449; slope with *P* < .05 nonzero). However, a significant increase in reconstruction techniques using tendon augmentation was observed during the study period (2013: n = 178; 2023: n = 292; R^2^: 0.70; y = 10.00x-19,942; F: 21.34; slope with *P* < .05 nonzero).

### Distribution of age

**Figure 3:**
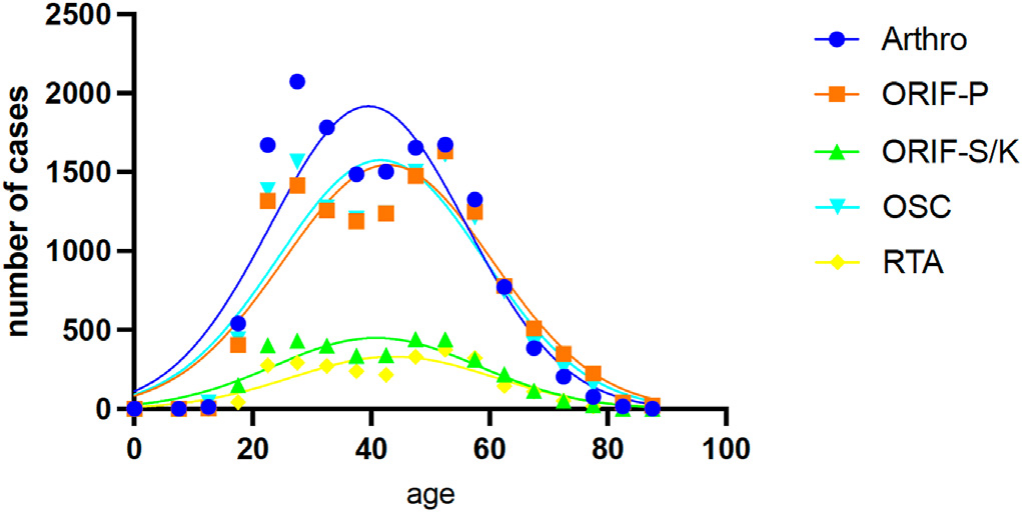
Gaussian regression analysis of the mean age of patients undergoing various surgical procedures for ACJD from 2013 to 2023. Mean ages and standard deviations are provided for each technique, emphasizing the preference for arthroscopic stabilization in younger individuals. *ACJD*, acromioclavicular joint dislocation; *Arthro*, arthroscopically assisted *ACJ* stabilization; ORIF-P, open reduction and internal fixation with plate; *ORIF-S/K*, open reduction and internal fixation with screws or Kirschner wires; *OSC*, open reconstruction using suture cerclage; *RTA*, reconstruction using tendon augmentation.

**Figure 4:**
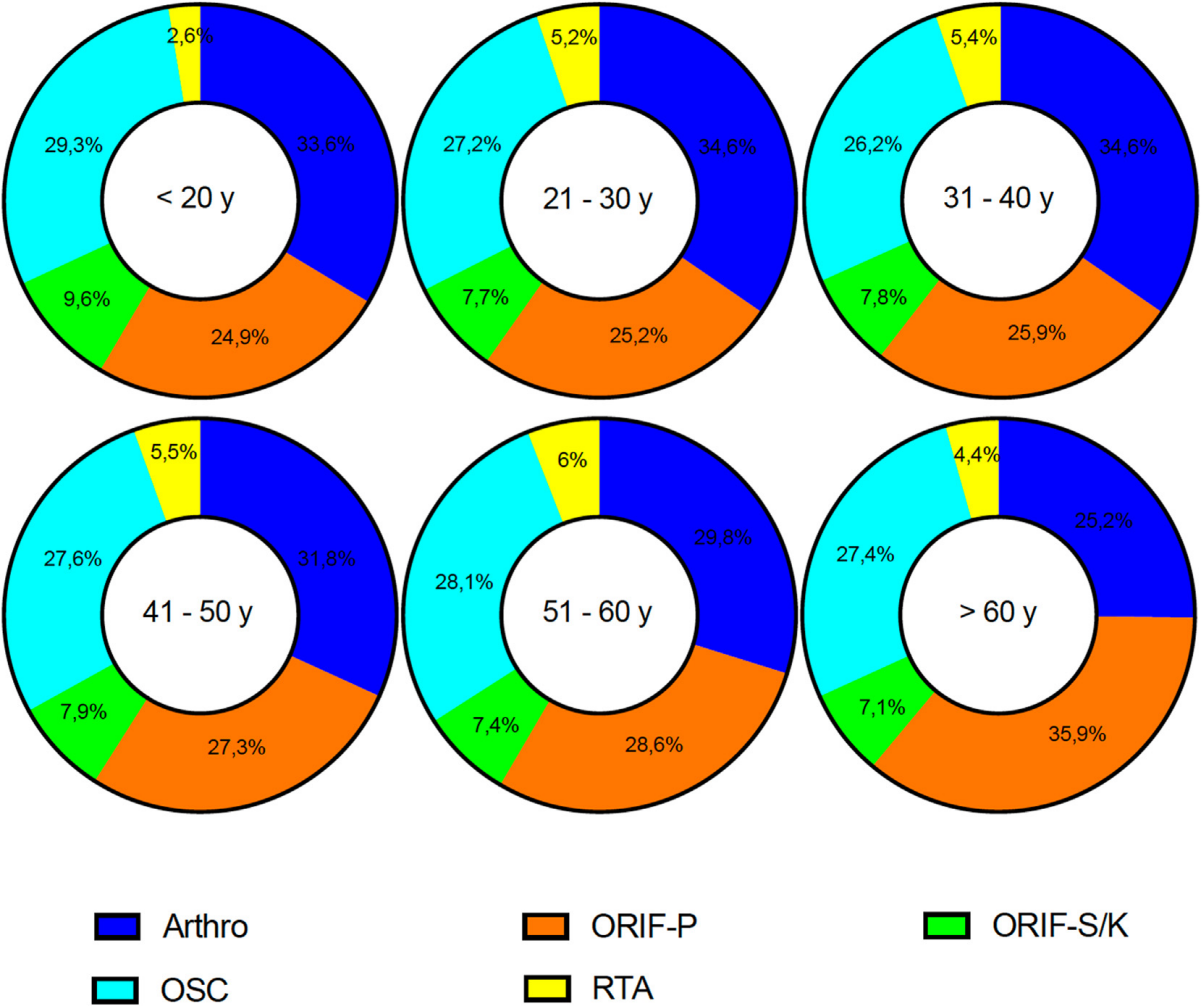
Age-stratified analysis of surgical techniques used for ACJD from 2013 to 2023. This categorization highlights age-specific preferences and the predominance of arthroscopic approaches in younger patients. Statistical analysis confirms significant associations between age groups and surgical techniques (*P* < .0001). *Arthro*, arthroscopically assisted ACJ stabilization; *ORIF-P*, open reduction and internal fixation with plate; *ORIF-S/K*, open reduction and internal fixation with screws or Kirschner wires; *OSC*, open reconstruction using suture cerclage; *RTA*, reconstruction using tendon augmentation.

The mean age of patients undergoing arthroscopically assisted stabilization (Arthro) was 39.6 ± 16.6 years, compared to 42.8 ± 17.7 years for ORIF-P, 40.9 ± 17.2 years for ORIF-S/K, 41.6 ± 17.4 years for OSC, and 43.8 ± 17.1 years for RTA. These findings indicate that the arthroscopic approach was predominantly utilized in a younger patient population, while reconstruction with additional tendon augmentation was most performed in the oldest age group (Fig. 3). Significant differences in the distribution of surgical techniques across various age groups were identified and statistically confirmed using the χ^2^ test. Among patients over 60 years of age, open surgical treatment with ORIF-P was the most frequently applied technique. In contrast, for all younger age subgroups (<20 years; 21-30 years; 31-40 years; 41-50 years; and 51-60 years), Arthro was the most used method. The age-specific distribution of surgical techniques is presented in Figure 4, highlighting the preference for different procedures based on age and emphasizing the shift toward arthroscopic methods in younger patients.

### Distribution of sex

**Figure 5:**
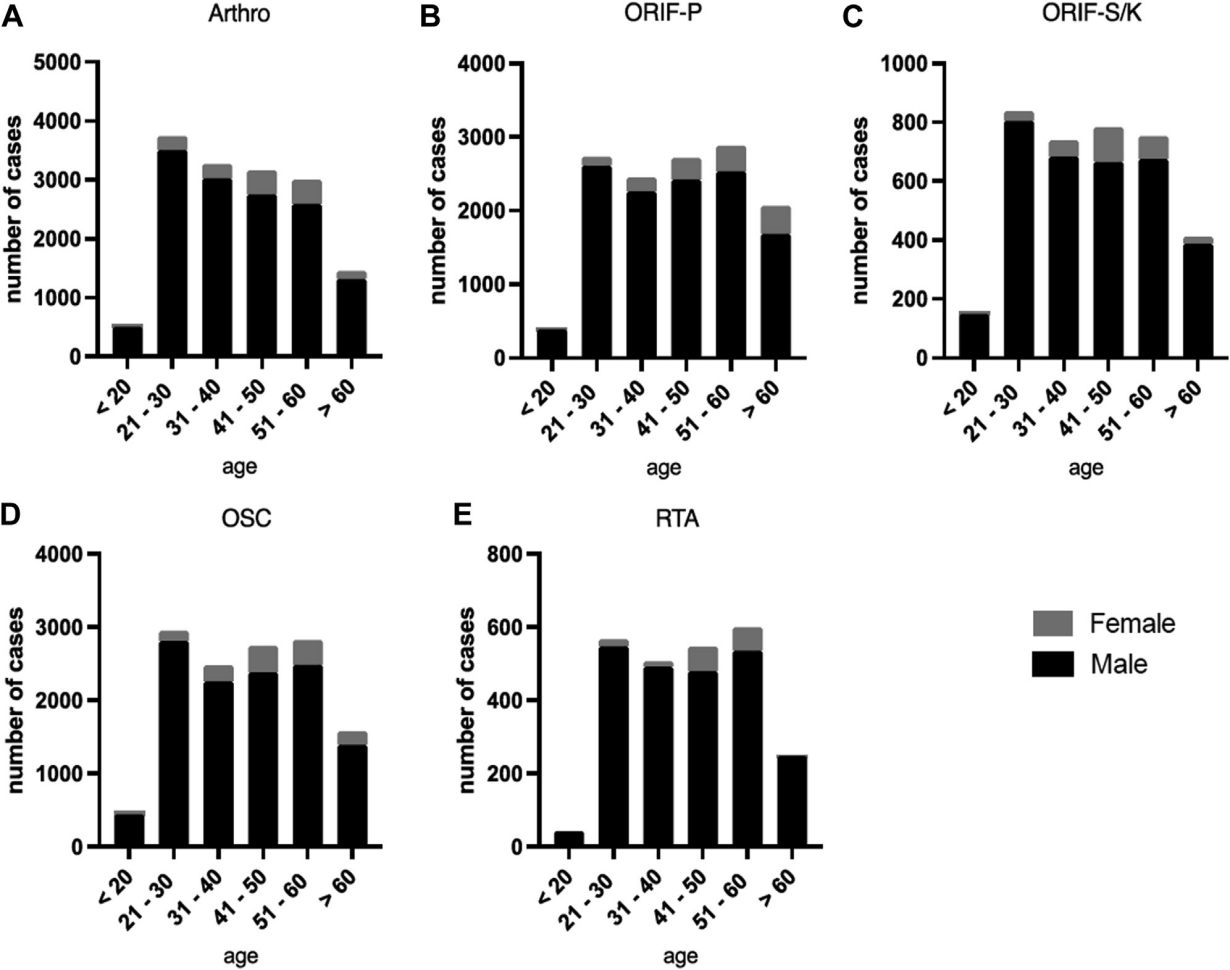
Sex-specific distribution of patients diagnosed with ACJD and treated surgically between 2013 and 2023. Data are stratified by surgical procedure and age, highlighting a significant male predominance across all techniques (*P* < .0001). *ACJD*, acromioclavicular joint dislocation; *Arthro*, arthroscopically assisted ACJ stabilization; *ORIF-P*, open reduction and internal fixation with plate; *ORIF-S/K*, open reduction and internal fixation with screws or Kirschner wires; *OSC*, open reconstruction using suture cerclage; *RTA*, reconstruction using tendon augmentation.

Analyzing the data across all age groups, the overall incidence of ACJD was 8.2 times higher in male than in female patients (f: n = 8941; m: n = 73,313) (Fig. 5). The highest incidence among women was observed in the “51-60” age group, with a male-to-female ratio of 6.9:1. The most pronounced sex-specific differences were observed across all surgical procedures, where the number of men treated consistently exceeded the number of women. In the most affected female age group, “51-60,” the number of men treated was 6.3 times higher for Arthro; 7.3 times higher for ORIF-P; 8.6 times higher for ORIF-S/K; 7.3 times higher for OSC; and 8.3 times higher for RTA. These differences were relatively consistent across the various surgical techniques. Figure 5 illustrates the sex distribution across the age groups for the diagnosis of ACJD and for all surgical procedures. These differences were statistically significant, as confirmed by the χ^2^ test.

## Discussion

This study provides a comprehensive analysis of trends in the management of hospitalized ACJD in Germany over the past decade, highlighting significant shifts in surgical approaches, demographic patterns, and numbers of hospitalized treated cases. The findings demonstrate a decent transition from conventional open surgical techniques, such as stabilization with plate and screws or Kirschner wire fixation, to arthroscopically assisted stabilization procedures. In Germany, inpatient hospitalization for ACJD is generally associated with surgical intervention, as conservative management is typically conducted on an outpatient basis. The decline in hospitalizations is therefore likely attributable to the increasing adoption of outpatient surgical procedures and the shift toward minimally invasive techniques, which may facilitate shorter hospital stays.

The observed decline in ACJD cases operated on at the hospital likely reflects broader changes in health care delivery practices and patient management strategies. One possible explanation is a trend toward conservative treatment in recent years for higher-grade Rockwood injuries, which were traditionally managed surgically.^5^^,^^7^^,^^8^^,^^22^^,^^24^^,^^28^^,^^33^^,^^41^^,^^42^ This trend aligns with emerging evidence questioning the necessity of surgical intervention for certain ACJD subtypes, particularly Rockwood type III injuries.^19^ As a result, fewer patients require hospitalization, as conservative management is typically conducted on an outpatient basis. This change has likely contributed significantly to the overall reduction in hospital admissions for ACJD. Moreover, the rise of minimally invasive surgical techniques, such as arthroscopically assisted stabilization, could also have further influenced the number of hospitalized treated cases. While arthroscopic procedures can still be billed as inpatient hospital services, significant numbers are performed in outpatient settings in Germany,^38^ which might be explained by potentially lower complication rates, faster recovery periods, advantages within the supply chain, standardized workflows, reduced exposure to nosocomial infections, and a lower risk of thrombosis.^1^^,^^25^^,^^40^ From 2015 to 2020, approximately 750,000 arthroscopic surgeries were conducted annually, accounting for about 4.65% of all inpatient procedures in Germany.^17^ Notably, in 2020, arthroscopic operations on knee articular cartilage and menisci, shoulder capsular ligament refixation and reconstruction, and synovial surgeries were among the 20 most commonly performed inpatient surgeries nationwide.^18^ This shift toward ambulatory care could also reflect broader trends in healthcare aiming to optimize resource utilization and cost reduction. Additionally, advancements in surgical techniques and perioperative management may have contributed to making outpatient arthroscopic procedures more feasible and safer,^20^ further supporting their increased adoption. As our dataset is derived from inpatient hospital billing data, outpatient surgical cases were not included in this study. Future research incorporating outpatient records would provide a more comprehensive understanding of ACJD treatment trends.

Another potential factor contributing to the decrease in hospitalized ACJD cases requiring surgery during the observed period could be the impact of the COVID-19 pandemic. During the COVID-19 pandemic, elective shoulder surgeries were postponed or canceled in many institutions, leading to a temporary decline in inpatient procedures.^26^^,^^36^^,^^39^ However, this period also facilitated an acceleration in the adoption of outpatient care pathways, including day-case arthroscopic surgeries.^21^ It remains unclear to what extent these pandemic-induced changes have persisted. Postpandemic, health care systems may have normalized to a hybrid model, combining an emphasis on outpatient care with a gradual resumption of inpatient services for more complex cases. The results of the present study demonstrate a general decline in hospital-based surgeries for ACJD between 2020 and 2021, followed by a subsequent increase in numbers starting in 2022. This trend reflects the previously reported reduction in hospital surgeries during the COVID-19 pandemic in Germany.^26^^,^^36^^,^^39^

The increasing reliance on less invasive arthroscopic techniques is a pivotal development in the surgical management of orthopedic conditions, including ACJD. This trend has notable implications for hospital stays, resource utilization, and procedural outcomes. One of the major advantages of arthroscopically assisted procedures over ORIF-P is the elimination of the need for a secondary intervention to remove osteosynthesis material. While surgery times for arthroscopic and open procedures are widely comparable,^16^ the minimally invasive approach may still lead to quicker postoperative recovery, reducing the duration of hospital stays and overall patient morbidity.^44^ Despite these advantages, arthroscopically assisted procedures come with significant technical demands. They require advanced arthroscopic skills and a higher degree of surgical expertise.^15^ The learning curve for mastering these techniques is steeper than for conventional open procedures, making them predominantly feasible in high-volume centers, university hospitals, and specialized orthopedic institutions.^3^^,^^4^ Peripheral hospitals, particularly in less urbanized areas, are likely to continue relying on traditional open techniques, as these require less specialized training and equipment. This shift toward minimally invasive techniques aligns with global trends in orthopedic surgery that prioritize patient safety, reduced invasiveness, and faster recovery. However, the adoption of these approaches remains uneven, reflecting disparities in resources and surgical expertise between larger, well-equipped centers, and smaller community-based hospitals.

The observed trend that arthroscopic stabilization is more frequently performed in younger patients (mean age: 39.6 years), while open reduction and internal fixation techniques, particularly plate stabilization, are predominantly utilized in older patients, represents an intriguing finding that warrants further exploration. This age-related preference for specific surgical techniques may not necessarily reflect inherent differences in clinical outcomes or functional recovery. Current evidence does not favor arthroscopic techniques over the conventional open procedures in terms of postoperative outcomes, return to sports, or return to daily activities.^14^^,^^27^^,^^34^ As such, the choice of surgical method may be influenced more by surgeon preference, training, or institutional protocols rather than by clear evidence of one technique's superiority. Although the differences between age groups were statistically significant, it is important to note that the patient population across all groups still primarily consists of relatively young individuals. Given that, the observed differences may not translate into a noticeable impact in routine clinical practice, as younger patients generally have better baseline recovery potential regardless of the surgical technique employed.^30^^,^^32^ However, this observation highlights the importance of further research to understand the factors driving these trends.

The significant male predominance observed in this study is consistent with previous literature, which attributes the higher incidence of ACJD among men to greater participation in high-risk physical activities and contact sports.^11^^,^^13^ However, the persistent sex-specific disparities in surgical management observed across all age groups, as highlighted by the authors' results, remain incompletely understood. Further research is required to determine whether these differences are driven by sociocultural influences, biological factors, or disparities in access to care.

The observed increase in tendon augmentation procedures for ACJD could reflect a growing recognition of this technique as a potential means to achieve additional biological stability in cases of chronic instability.^9^ This trend might suggest a shift in surgical practice, with surgeons possibly becoming more willing to address chronic ACJD using tendon augmentation, often in combination with arthroscopically assisted techniques. While arthroscopic stabilization is predominantly performed in acute ACJD cases, its application in chronic instability cases—in combination with tendon augmentation—has been increasingly reported.^37^ However, the inability to differentiate between acute and chronic cases using OPS codes remains a limitation of this study. From a biomechanical standpoint, tendon augmentation is particularly beneficial in chronic cases, where the native ligament structures have undergone degeneration and atrophy, necessitating biological support.^29^^,^^31^ However, while RTA is predominantly used in chronic cases, its application in select acute cases prevents definitive classification based solely on OPS coding. Thus, although RTA utilization strongly suggests chronic injury, absolute confirmation is not possible. This highlights a limitation in the current OPS classification system, which does not allow for precise differentiation between acute and chronic cases, potentially impacting the interpretation of the results. In chronic cases, degeneration and atrophy of the coraco-clavicular and acromioclavicular ligaments necessitate incorporation of biological augmentation alongside mechanical fixation. Common approaches for biological support include coracoacromial ligament transfer or the use of a tendon graft.^10^^,^^12^^,^^43^ In earlier years, such complex cases may have been more commonly managed conservatively, as surgical intervention for chronic instability was less frequently pursued. These changes could indicate an evolving approach addressing complex ACJD cases, with an increasing preference for innovative surgical solutions over traditional nonoperative methods. However, a limitation to this explanation is the inability to code chronic ACJD as a distinct entity within the ICD-10 classification system. This makes it challenging to conduct a precise analysis of the trends and developments in the use of tendon augmentations for surgical chronic ACJD stabilization. Future studies should further differentiate tendon augmentation techniques used in ACJD treatment, including autograft, allograft, synthetic, or bioinductive materials, to better understand their impact on clinical outcomes.

This study is not without limitations. The retrospective design limits the ability to establish causality, and reliance on hospital billing data may not capture all nuances, particularly for outpatient or conservatively treated cases. It is important to note that the hospital billing data used in this study exclusively include patients who were admitted for surgical intervention. Outpatient cases and non-operative treatments were not captured in this dataset, potentially underestimating the total number of ACJD cases. Additionally, the absence of specific ICD-10 codes for chronic ACJD complicates detailed analyses of tendon augmentation trends. Variations in surgeon expertise and institutional protocols, as well as the lack of patient-reported outcomes or functional data, further restrict the interpretation of findings and their correlation with clinical efficacy. OPS codes provide a general classification of surgical interventions but do not specify exact techniques, devices, or graft usage. Thus, further stratification was not possible within the scope of this study. Despite these limitations, this study provides novel and high-quality data on national trends in ACJD management over the last decade. By offering a comprehensive analysis of demographic and procedural patterns, our findings deliver valuable insights to guide clinical decision-making and improve treatment strategies. These results represent a significant contribution to understanding and advance of evidence-based approaches in ACJD care.

## Conclusion

Over the past decade, the management of hospitalized patients with ACJDs in Germany has undergone significant changes. The number of hospitalized treated cases has declined, while arthroscopically assisted stabilization has emerged as the predominant surgical technique, particularly in younger patients. In contrast, traditional open techniques, such as plate stabilization and screw or Kirschner wire fixation, have decreased substantially. Demographic analysis revealed a strong male predominance and an age-related preference for specific surgical approaches. These findings highlight evolving trends in surgical management and underscore the increasing adoption of minimally invasive techniques for ACJDs.

## Declaration of generative AI and AI-assisted technologies in the writing process

During the preparation of this work, the authors used ChatGPT (version January 2024, OpenAI) to refine language and improve clarity in scientific communication. After using this service, the authors reviewed and edited the content as needed and take full responsibility for the content of the publication.

## Disclaimers

Funding: No funding was disclosed by the authors.

Conflicts of interest: Kilian List is a consultant for Arthrex and Stryker. The other authors, their immediate families, and any research foundation with which they are affiliated have not received any financial payments or other benefits from any commercial entity related to the subject of this article. None of the authors has received any financial remuneration related to the subject of the article.
